# Extensively Drug-Resistant Tuberculosis, China

**DOI:** 10.3201/eid1703.100919

**Published:** 2011-03

**Authors:** Shenjie Tang, Qing Zhang, Jinming Yu, Yidian Liu, Wei Sha, Hua Sun, Lin Fan, Jin Gu, Xiaohui Hao, Lan Yao, Heping Xiao

**Affiliations:** Author affiliations: Shanghai Pulmonary Hospital, Tongji University School of Medicine, Shanghai, People’s Republic of China (S. Tang, Q. Zhang, J. Yu, Y. Liu, W. Sha, H. Sun, L. Fan, J. Gu, X. Hao, L. Yao, H. Xiao);; Shanghai Key Laboratory of Tuberculosis, Shanghai (S. Tang, Q. Zhang, J. Yu, Y. Liu, W. Sha, H. Sun, L. Fan, J. Gu, X. Hao, L. Yao, H. Xiao);; Fudan University School of Public Health, Shanghai (J. Yu)

**Keywords:** Extensively drug-resistant tuberculosis, bacteria, antimicrobial resistance, tuberculosis and other mycobacteria, drug susceptibility testing, China, letter

**To the Editor:** The prevalence of drug-resistant tuberculosis (TB) is a serious problem in the People’s Republic of China. China is 1 of 22 countries with the highest incidence of TB (*1*). It is also 1 of 27 countries with the highest incidence of multidrug-resistant TB (MDR TB) and extensively drug-resistant TB (XDR TB). According to the national baseline survey on TB in 2007 and 2008, the frequency of MDR TB among pulmonary TB patients in China was 8.3%. We estimate that there are 120,000 new cases of MDR TB in China per year, which accounts for 24.0% of new cases worldwide (510,000) per year.

XDR TB has recently emerged as a global public health problem (*2*). It is defined as TB with resistance to at least isoniazid, rifampin, a fluoroquinolone, and 1 of 3 injectable second-line drugs (amikacin, kanamycin, or capreomycin). XDR TB is a type of MDR TB that shows resistance to isoniazid and rifampin. Recent reports on current prevalence of XDR TB (*3**,**4*) indicate that China now has the second highest incidence of MDR TB worldwide. However, there is no information available on XDR TB in China.

To obtain information on XDR TB in China, we conducted a study at Shanghai Pulmonary Hospital. It is the only specialized hospital for TB in Shanghai and plays a major role in treating TB patients and providing state-of- the-art treatment. Most patients referred to this hospital have been previously treated or have recurrent TB. Therefore, higher rates of MDR TB and XDR TB are expected in this setting, which is not comparable to community or multicenter-based studies.

Patients with culture-proven MDR TB during January 2008–June 2009 were retrospectively evaluated. All patients were HIV negative. Drug susceptibility testing was conducted for culture-positive isolates by using the BACTEC 960 System (Becton Dickinson, Franklin Lakes, NJ, USA) at concentrations of 0.1 μg/mL for isoniazid, 1 μg/mL for rifampin, 5 μg/mL for ethambutol, 1 μg/mL for streptomycin, 2.5 μg/mL for capreomycin, 1μg/mL for amikacin, and 2 μg/mL for ofloxacin.

Among 518 strains that were culture positive for *Mycobacterium tuberculosis*, 350 (67.6%) were drug resistant and 168 (32.4%) were drug sensitive. A total of 217 (41.9%) of 518 strains were classified as MDR and accounted for 62.0% of drug-resistant strains. Among 217 MDR strains, 45 (20.7%) were from patients who had a new diagnosis of TB, and 172 (79.3%) were from patients whose medical history included treatment for TB for >4 weeks. A total of 65 (12.6%) strains were XDR, of which 51 were from patients previously treated. These strains accounted for 18.6% of drug-resistant strains and 30.0% of MDR strains.

Of 217 MDR isolates, 217 (100.0%), 217 (100.0%), 172 (79.3%), 175 (80.6%), 170 (78.3%), 68 (31.3%), and 69 (31.8%) were resistant to isoniazid, rifampicin, streptomycin, ethambutol, ofloxacin, capreomycin, and amikacin, respectively. Of 65 XDR isolates, 65 (100.0%), 65 (100.0%), 61 (93.9%), 60 (92.3%), 65 (100.0%), 60 (92.3%), and 60 (92.3%) were resistant to isoniazid, rifampicin, streptomycin, ethambutol, ofloxacin, capreomycin, and amikacin, respectively.

Our results indicate that 30.0% of MDR strains were XDR strains. Although our study was conducted in only 1 hospital, this prevalence of XDR strains indicates that XDR TB in China is a serious concern. A total of 78.3% of MDR isolates were resistant to ofloxacin, which is higher than rates reported for South Korea (42.8%) (*5*) and Taiwan (16.6%) (*6*). Population-based studies have reported lower frequencies of XDR strains among MDR strains; 9.9% for 14 qualified reference laboratories (*7*), 5.3% for South Korea (*8*), and 23.9% for South Africa among patients co-infected with HIV and TB (*9*).

In our study, 2 factors may have contributed to high drug-resistance rates. First, fluoroquinolones have been widely used for treatment of respiratory tract bacterial infections because of their efficacy and mild adverse reactions. Second, we also prescribed fluoroquinolones for treatment of patients with drug-resistant TB and some patients with drug-sensitive TB who could not tolerate first-line anti-TB drugs. More than 90% of patients with XDR TB had strains resistant to streptomycin, ethambutol, capreomycin, and amikacin, which was higher than rates reported in other studies (*5**,**9**,**10*). Currently, anti-TB medications in China for treatment of patients with XDR TB are scarce. This scarcity has resulted in poor treatment outcomes in patients with XDR TB.

One limitation of our study is that we investigated patients at only 1 specialized TB hospital in Shanghai. Therefore, data are not representative for the general population. A community-based multicenter study is needed to determine the true prevalence of XDR TB in China. Nevertheless, our study confirms that the prevalence of MDR TB and XDR TB is high in some areas. It also emphasizes the need to increase TB prevention and therapy, educate society about TB, implement modern TB control strategies, and strengthen basic and clinical research to curb the spread of MDR TB and XDR TB.
